# Supplementary figures and images for: ATXN3 regulates lysosome regeneration after damage by targeting K48-K63-branched ubiquitin chains
Source: EMBO J. 2025 Jul 29;44(18):5086–111. doi: 10.1038/s44318-025-00517-x (PMC12436607; doi:10.1038/s44318-025-00517-x)

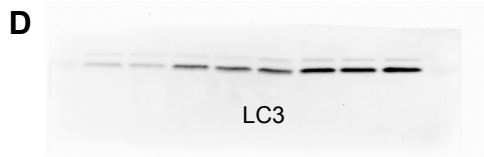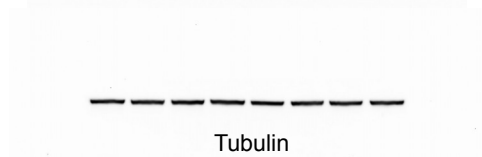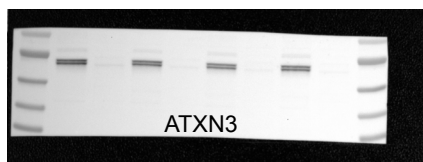

Fig. 3

Supplement: Supplementary file 4 — Source data Fig. 3 [file 44318_2025_517_MOESM4_ESM.zip › Figure 3/WholeScans_Fig3.pdf]

**G**

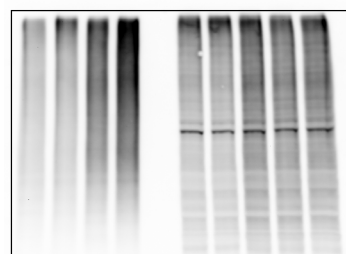

Ubiquitin  
(P4D1)

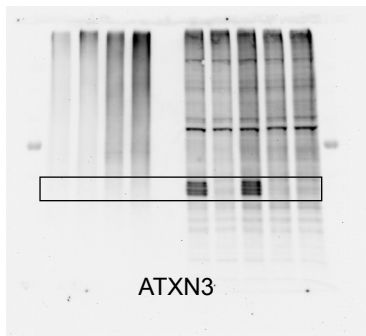

ATXN3

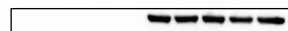

Tubulin

Supplement: Supplementary file 6 — Source data Fig. 5 [file 44318_2025_517_MOESM6_ESM.zip › Figure5/WholeScans_Fig5.pdf]

**B**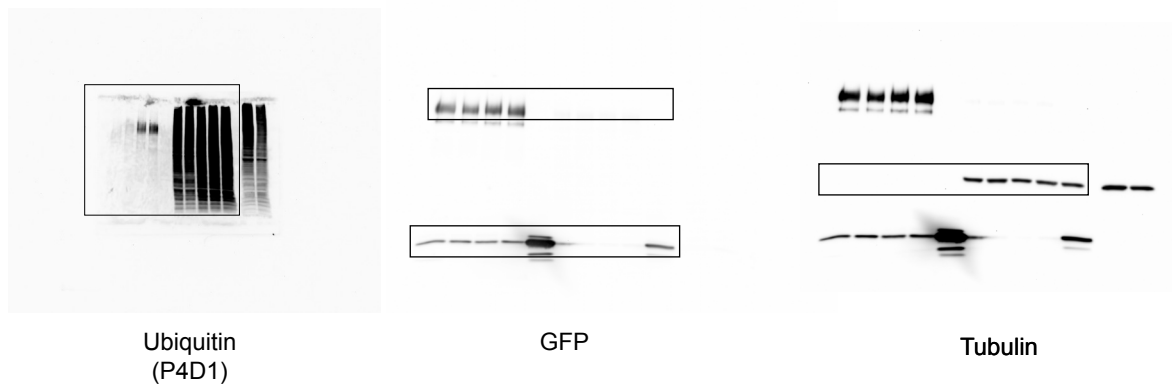**C**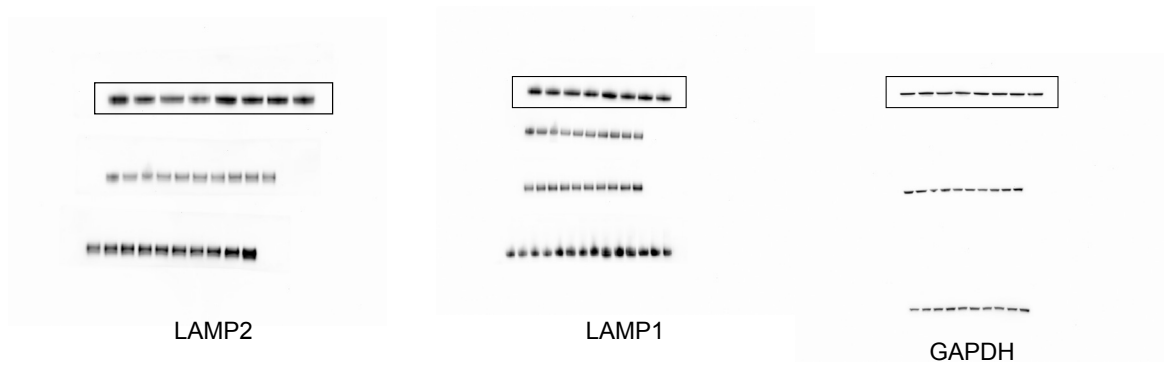**E**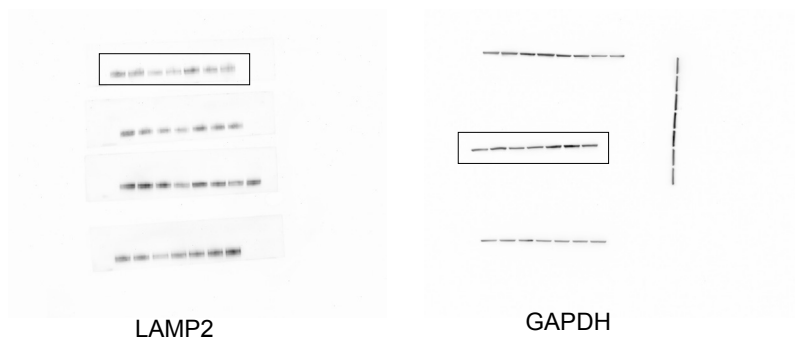

Fig. 6

Supplement: Supplementary file 7 — Source data Fig. 6 [file 44318_2025_517_MOESM7_ESM.zip › Figure 6/WholeScans_Fig6.pdf]
