# Supplementary material for: ATXN3 regulates lysosome regeneration after damage by targeting K48-K63-branched ubiquitin chains
Source: EMBO J. 2025 Jul 29;44(18):5086–111. doi: 10.1038/s44318-025-00517-x (PMC12436607; doi:10.1038/s44318-025-00517-x)
Supplement: Supplementary file 8 — Expanded View Figures [file 44318_2025_517_MOESM8_ESM.pdf]

Expanded View Figures

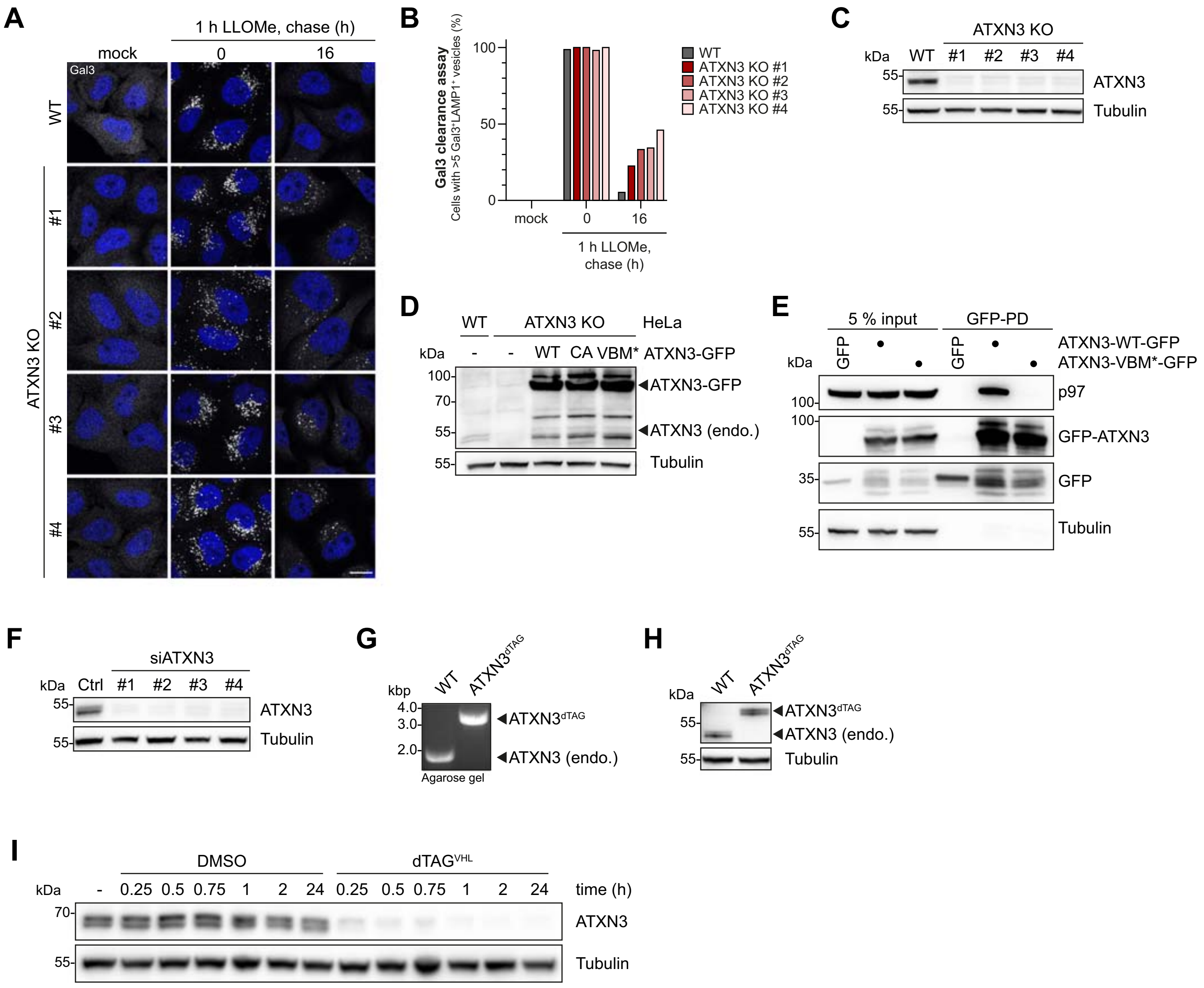

Figure EV1. (related to Fig. 2): ATXN3 is essential for the clearance of damaged lysosomes and the restoration of degradative compartments after lysosome damage.

(**A**) Gal3 clearance assays in HeLa parental and 4 different ATXN3 KO clones performed as in Fig. 2A. Scale bar, 15 µm. (**B**) Quantification of (**A**), representative experiment with >40 cells per conditions. The graph shows the mean. (**C**) Western blot verification of ATXN3 KO. (**D**) Western blot verification of rescue constructs used in Fig. 2A. (**E**) ATXN3 mutant of the VBM motif (VBM*) is deficient in p97 binding. (**F**) Western blot evaluation of siRNA-mediated ATXN3 depletion. (**G**) PCR on genomic DNA confirming genomic insertion of the FKBP^{F36V} tag in ATXN3^{dTAG} U2OS cells. (**H**) Western blot analysis of lysates of U2OS WT and gene-edited ATXN3^{dTAG} cells. (**I**) Western blot assessment of the induced degradation of ATXN3 ^{dTAG} in U2OS cells.

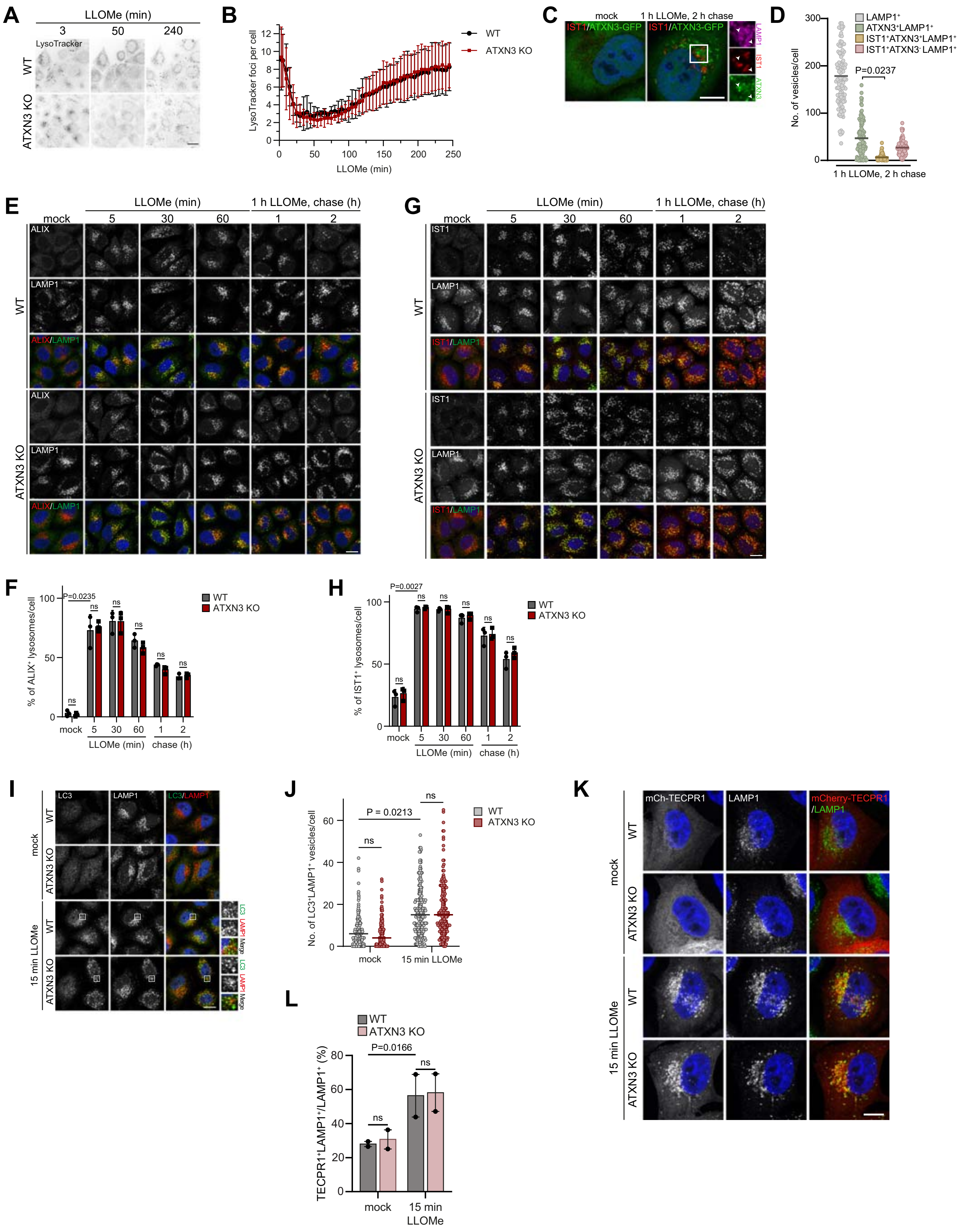
A
LLOMe (min)
3
50
240
LysoTracker
WT
ATXN3 KO
B
LysoTracker foci per cell
LLOMe (min)
WT
ATXN3 KO
C
mock
1 h LLOMe, 2 h chase
IST1/ATXN3-GFP
LAMP1
IST1
ATXN3
D
No. of vesicles/cell
LAMP1+
ATXN3+LAMP1+
IST1+ATXN3+LAMP1+
IST1+ATXN3-LAMP1+
P=0.0237
1 h LLOMe, 2 h chase
E
mock
LLOMe (min)
5
30
60
1 h LLOMe, chase (h)
1
2
ALIX
LAMP1
ALIX/LAMP1
WT
ATXN3 KO
G
IST1
LAMP1
IST1/LAMP1
F
% of ALIX+ lysosomes/cell
P=0.0235
ns
WT
ATXN3 KO
LLOMe (min)
chase (h)
H
% of IST1+ lysosomes/cell
P=0.0027
I
LC3
LAMP1
LC3/LAMP1
mock
15 min LLOMe
Merge
J
No. of LC3+LAMP1+ vesicles/cell
P = 0.0213
15 min LLOMe
K
mCh-TECPR1
mCherry-TECPR1/LAMP1
L
TECPR1+LAMP1+/LAMP1+ (%)
P=0.0166

Figure EV2. ATXN3 is not involved in initial membrane repair pathways after lysosomal damage.

(**A**) HeLa WT and ATXN3 KO cells were loaded with LysoTracker, treated with LLOMe, and LysoTracker recovery was imaged over the indicated period. Note that recovery of LysoTracker is unaffected in ATXN3 KO cells. Scale bar, 20 µm. (**B**) Quantification of (**A**), $n = 3$ biologically independent experiments with >70 cells quantified per condition per experiment. The graph shows mean ± SD. (**C**) Lack of colocalisation of ATXN3 and the ESCRT-III component IST1. HeLa cells expressing ATXN3-GFP were treated as indicated, fixed, and immuno-stained for IST1 and LAMP1. Arrowheads indicate ATXN3-positive and IST1-negative lysosomes. Scale bar, 10 µm. (**D**) Quantification of (**C**). $n = 3$ biological replicates with >30 cells per condition per experiment. One-way ANOVA with Tukey's multiple comparison test. The line indicates the mean. (**E**) ALIX localization is not affected in ATXN3 KO cells. HeLa WT or ATXN3 KO cells were mock- or LLOMe-treated and stained with the indicated antibodies. Scale bar, 15 µm. (**F**) Quantification of (**E**). $n = 3$ biological independent experiments with >40 cells per condition per experiment. Significance was tested by two-way ANOVA with Dunnett's multiple comparison test. The graph shows mean ± SD. (**G**) IST1 localization is not changed in HeLa ATXN3 KO compared to WT cells. Cells were treated as indicated, fixed, and immuno-stained for IST1 and LAMP1. Scale bar, 15 µm. (**H**) Quantification of (**G**). $n = 3$ biological replicates with >40 cells per condition per experiment. Two-way ANOVA with Dunnett's multiple comparison test. The graph shows mean ± SD. (**I**) Early LC3 recruitment indicates that CASM is not affected in ATXN3 KO cells. HeLa WT or ATXN3 KO cells were treated for 15 min with LLOMe and stained with the indicated antibodies. Scale bar, 15 µm. (**J**) Quantification of (**I**). $n = 3$ biologically independent experiments with >40 cells per condition per experiment. Significance was tested by two-way ANOVA with Fisher's LSD test. The graph shows mean ± SD. (**K**) Recruitment of TECPR1 is not compromised in ATXN3 KO cells. HeLa WT or ATXN3 KO cells transiently overexpressing mCherry-TECPR1 were mock- or LLOMe-treated for LLOMe for 15 min and stained for LAMP1. Scale bar, 10 µm. (**L**) Quantification of (**K**). $n = 2$ biologically independent experiments with 18 or more cells per condition per experiment. Two-way ANOVA with uncorrected Fisher's LSD test. The graph shows mean ± SEM.

© The Author(s)

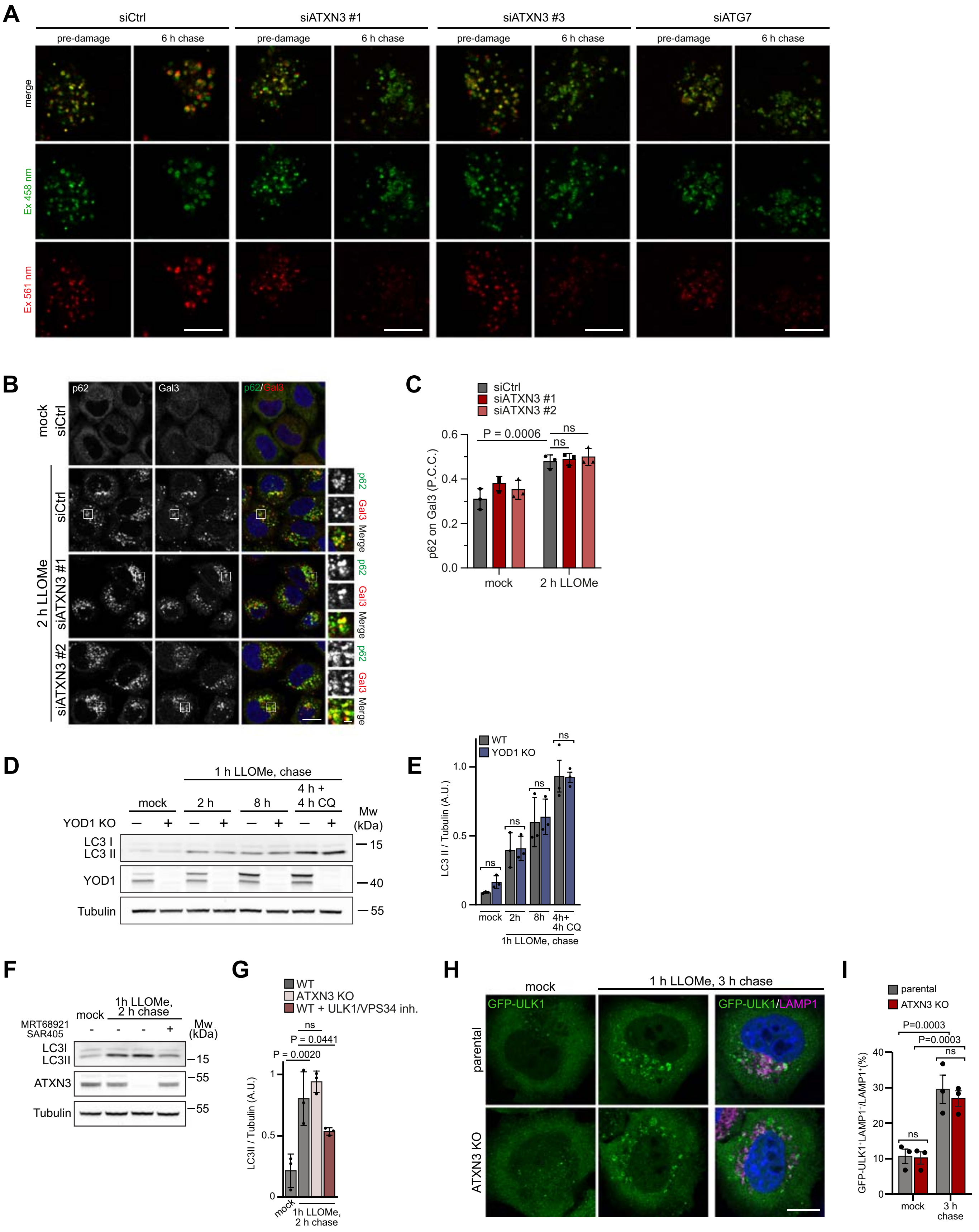
A
siCtrl
siATXN3 #1
siATXN3 #3
siATG7
pre-damage
6 h chase
merge
Ex 458 nm
Ex 561 nm
B
p62
Gal3
p62/Gal3
mock siCtrl
2 h LLOMe
siCtrl
siATXN3 #1
siATXN3 #2
Merge
C
siCtrl
siATXN3 #1
siATXN3 #2
P = 0.0006
ns
p62 on Gal3 (P.C.C.)
0.0
0.2
0.4
0.6
mock
2 h LLOMe
D
1 h LLOMe, chase
mock
2 h
8 h
4 h +
4 h CQ
YOD1 KO
Mw
(kDa)
LC3 I
LC3 II
YOD1
Tubulin
15
40
55
E
WT
YOD1 KO
ns
LC3 II / Tubulin (A.U.)
0
0.5
1.0
mock
2h
8h
4h+
4h CQ
1h LLOMe, chase
F
1h LLOMe,
2 h chase
mock
MRT68921
SAR405
Mw
(kDa)
LC3I
LC3II
ATXN3
Tubulin
15
55
G
WT
ATXN3 KO
WT + ULK1/VPS34 inh.
ns
P = 0.0441
P = 0.0020
LC3II / Tubulin (A.U.)
0
0.5
1
mock
1h LLOMe,
2 h chase
H
mock
1 h LLOMe, 3 h chase
GFP-ULK1
GFP-ULK1/LAMP1
parental
ATXN3 KO
I
parental
ATXN3 KO
P=0.0003
P=0.0003
ns
GFP-ULK1+LAMP1+/LAMP1+(%)
0
10
20
30
40
mock
3 h
chase

 © The Author(s)

Figure EV3. (related to Fig. 3) ATXN3 is required for the completion of lysophagy.

(**A**) Micrographs corresponding to the TMEM192-mKeima lysophagy assay in Fig. 3A as indicated. Scale bar, 10 µm. (**B**) ATXN3 depletion does not affect recruitment of SQSTM1/p62 to damaged lysosomes. HeLa cells treated with indicated siRNAs were mock or LLOMe-treated and fixed after 2 h. Cells were immuno-stained for Gal3 and p62. Note that these samples were also stained for LC3 (shown in Fig. 3B). Scale bar, 15 µm. (**C**) Quantification of (**B**), $n = 3$ biologically independent experiments with >70 cells quantified per condition per experiment. Two-way ANOVA with Tukey's multiple comparison test. The graph shows mean ± SD. (**D**) LC3 lipidation is not affected in YOD1 KO cells. (**E**) Quantification of (**D**). $n = 3$ biologically independent experiments. Two-way ANOVA with Tukey's multiple comparison test. Error bars represent the mean with SD. (**F**) Phagophore-associated LC3 lipidation during the lysosomal damage response is not affected by ATXN3 KO. Phagophore formation was inhibited by the indicated ULK1/2 and PI3KC3 inhibitors, and LC3-II formation was detected by Western blot analysis. Note the reduction of LC3-II after inhibition of phagophore formation, which is not observed in ATXN3 KO cells. (**G**) Quantification of (**F**). $n = 3$ biologically independent experiments. One-way ANOVA with Newman–Keuls multiple comparison test. Error bars represent the mean with SD. (**H**) ULK1 translocation to damaged lysosomes is not affected in ATXN3 KO cells. Micrographs show wildtype and ATXN3 KO cells transiently overexpressing GFP-ULK1 in control conditions and after 1 h LLOMe followed by 3 h chase. Scale bar, 10 µm. (**I**) Quantification of (**H**). $n = 3$ biologically independent experiments with >15 cells per experiment. Two-way ANOVA with Tukey's multiple comparison test. Error bars represent the mean with SEM.

© The Author(s)

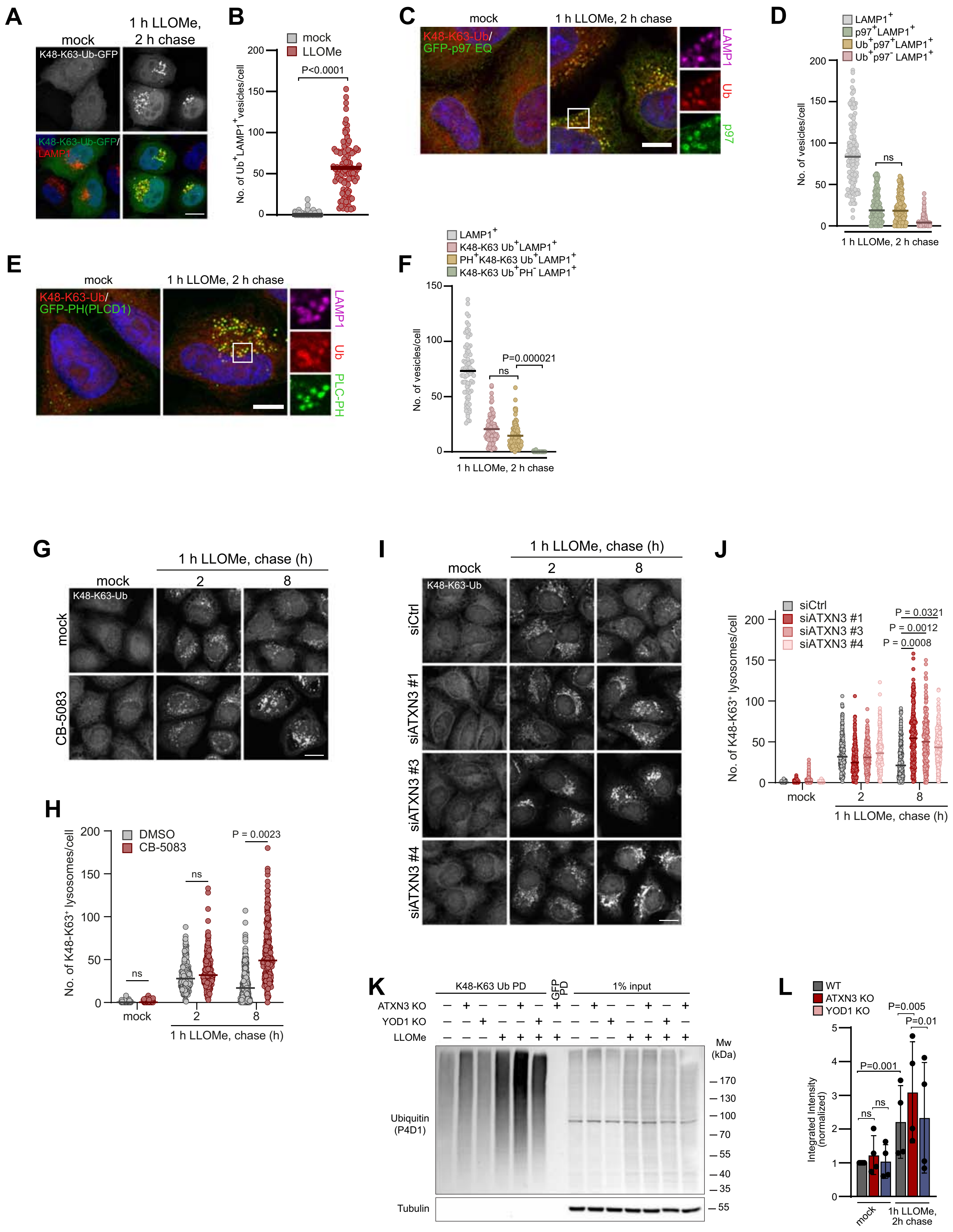
A
mock
1 h LLOMe, 2 h chase
K48-K63-Ub-GFP
K48-K63-Ub-GFP/ LAMP1
B
mock
LLOMe
P<0.0001
No. of Ub+LAMP1+ vesicles/cell
C
mock
1 h LLOMe, 2 h chase
K48-K63-Ub/ GFP-p97 EQ
LAMP1
Ub
p97
D
LAMP1+
p97+LAMP1+
Ub+p97+LAMP1+
Ub+p97- LAMP1+
ns
No. of vesicles/cell
1 h LLOMe, 2 h chase
E
mock
1 h LLOMe, 2 h chase
K48-K63-Ub/ GFP-PH(PLCD1)
LAMP1
Ub
PLC-PH
F
LAMP1+
K48-K63 Ub+LAMP1+
PH+K48-K63 Ub+LAMP1+
K48-K63 Ub+PH- LAMP1+
P=0.000021
ns
No. of vesicles/cell
1 h LLOMe, 2 h chase
G
1 h LLOMe, chase (h)
mock
2
8
K48-K63-Ub
mock
CB-5083
H
DMSO
CB-5083
P = 0.0023
ns
No. of K48-K63+ lysosomes/cell
mock
1 h LLOMe, chase (h)
I
1 h LLOMe, chase (h)
mock
2
8
K48-K63-Ub
siCtrl
siATXN3 #1
siATXN3 #3
siATXN3 #4
J
siCtrl
siATXN3 #1
siATXN3 #3
siATXN3 #4
P = 0.0321
P = 0.0012
P = 0.0008
No. of K48-K63+ lysosomes/cell
mock
1 h LLOMe, chase (h)
K
K48-K63 Ub PD
GFP PD
1% input
ATXN3 KO
YOD1 KO
LLOMe
Mw (kDa)
170
130
100
70
55
40
35
55
Ubiquitin (P4D1)
Tubulin
L
WT
ATXN3 KO
YOD1 KO
P=0.005
P=0.01
P=0.001
ns
ns
Integrated Intensity (normalized)
mock
1h LLOMe, 2h chase

© The Author(s)

Figure EV4. (related to Fig. 5). Damaged lysosomes are modified with K48-K63-branched ubiquitin chains.

(**A**) HeLa cells expressing NbSL3.3Q-GFP specific for K48-K63-branched chains were mock or LLOMe-treated, fixed, and immuno-stained for LAMP1. Note the prominent localization of NbSL3.3Q-GFP on lysosomes specifically after induction of lysosome damage. Scale bar, 15 µm. (**B**) Quantification of (**A**), $n = 3$ biological replicates with >20 cells per condition per experiment. Statistical significance was calculated with an unpaired *t*-test. Lines represent the mean. (**C**) HeLa cells expressing p97-E578Q were fixed and stained for K48-K63-branched ubiquitin chains with NbSL3.3Q. Scale bar, 10 µm. (**D**) Quantification of (**C**), $n = 3$ biological replicates with >30 cells per condition per experiment. One-way ANOVA with Tukey's multiple comparison test. The line indicates the mean. (**E**) K48-K63 branched chains accumulate on regenerating lysosomes. Scale bar, 10 µm. (**F**) Quantification of (**E**). $n = 2$ biologically independent experiments with >30 cells per condition per experiment. Lines represent the mean. Unpaired student's *t*-test. (**G**) HeLa cells were incubated with p97 inhibitor CB-5083 or vehicle alone and mock or LLOMe-treated. Cells were fixed at indicated time points and stained with NbSL3.3Q for K48-K63-branched ubiquitin chains. Scale bar, 15 µm. (**H**) Quantification of (**E**) $n = 3$ biological replicates with >70 cells per condition per experiment. Two-way ANOVA with Tukey's multiple comparison test. The line indicates the median. (**I**) Damage-induced K48-K63-branched chains persist in ATXN3-depleted cells. HeLa cells were treated with indicated siRNAs and K48-K63-branched chains persistence was monitored after lysosomal damage was assayed as in (**G**). Scale bar, 15 µm. (**J**) Quantification of (**G**). $n = 3$ biologically independent experiments with >30 cells quantified per condition per experiment. Two-way ANOVA with Tukey's multiple comparison test was used to test significance. The line indicates the median. (**K**) Damage-induced K48-K63-branched chains accumulate in ATXN3 KO cells, but not in YOD1 KO cells. (**L**) Quantification of (**I**). $n = 4$ biologically independent experiments. Two-way ANOVA with Uncorrected Fisher's LSD test. Error bars represent the mean with SD.

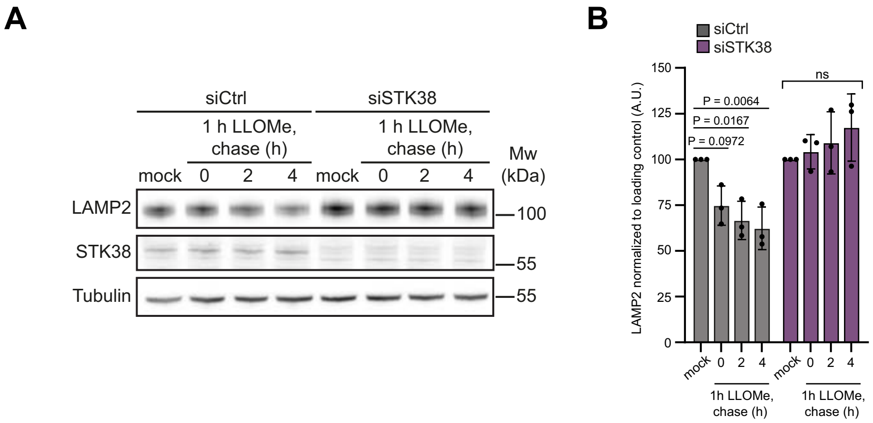

Figure EV5. (related to Fig. 6). LAMP2 is degraded through STK38-regulated microautophagy.

(**A**) Knockdown of STK38 inhibits LAMP2 degradation upon induction of lysosomal damage. HeLa Kyoto cells were transfected with non-coding siRNA or siRNA against STK38. 48 h post-transfection, cells were either mock- or LLOMe-treated and chased for the indicated time. (**B**) Quantification of (**A**). $n = 3$ biologically independent experiments. Two-way ANOVA with Sidak's multiple comparison test. Error bars represent the mean with SD.

 © The Author(s)
